# miR-133 regulates Evi1 expression in AML cells as a potential therapeutic target

**DOI:** 10.1038/srep19204

**Published:** 2016-01-12

**Authors:** Haruna Yamamoto, Jun Lu, Shigeyoshi Oba, Toyotaka Kawamata, Akihide Yoshimi, Natsumi Kurosaki, Kazuaki Yokoyama, Hiromichi Matsushita, Mineo Kurokawa, Arinobu Tojo, Kiyoshi Ando, Kazuhiro Morishita, Koko Katagiri, Ai Kotani

**Affiliations:** 1 Department of Hematological Malignancy, Institute of Medical Science, Tokai University, 143 Shimokasuya, Isehara, Kanagawa 259-1193 Japan; 2 Department of Intractable Diseases, Institute of National Center for Global Health and Medicine, 1-21-1 Toyama, Shinjuku-ku, Tokyo 162-8655 Japan; 3 Department of Nephrology and Endocrinology, the University of Tokyo Hospital, 7-3-1 Hongo, Bunkyo-ku, Tokyo 113-8655 Japan; 4 Department of Hematology/ Oncology, Institute of Medical Science, University of Tokyo, 4-6-1 Shirokanedai, Minato-ku, Tokyo 108-8639 Japan; 5 Department of Hematology and Oncology, Graduate School of Medicine, the University of Tokyo, 4-6-1 Shirokanedai, Minato-ku, Tokyo 108-8639 Japan; 6 Department of Hematology / Oncology, School of Medicine, Tokai University, 143 Shimokasuya, Isehara, Kanagawa 259-1193 Japan; 7 Department of Medical Science, Faculty of Medicine, University of Miyazaki, 5200 Kiyotakecho Kihara, Miyazaki-city, Miyazaki 889-1692 Japan; 8 Department of Biosciences, School of Science, Kitasato University 1-15-1, Kitasato, Minami-ku, Sagamihara, Knagawa 252-0373 Japan; 9 Japan Science and Technology Agency (JST) Science Plaza 5-3, Yonbancho, Chiyoda-ku, Tokyo 102-8666 Japan

## Abstract

The Ecotropic viral integration site 1 (Evi1) is a zinc finger transcription factor, which is located on chromosome 3q26, over-expression in some acute myeloid leukemia (AML) and myelodysplastic syndrome (MDS). Elevated Evi1 expression in AML is associated with unfavorable prognosis. Therefore, Evi1 is one of the strong candidate in molecular target therapy for the leukemia. MicroRNAs (miRNAs) are small non-coding RNAs, vital to many cell functions that negatively regulate gene expression by translation or inducing sequence-specific degradation of target mRNAs. As a novel biologics, miRNAs is a promising therapeutic target due to its low toxicity and low cost. We screened miRNAs which down-regulate Evi1. miR-133 was identified to directly bind to Evi1 to regulate it. miR-133 increases drug sensitivity specifically in Evi1 expressing leukemic cells, but not in Evi1-non-expressing cells The results suggest that miR-133 can be promising therapeutic target for the Evi1 dysregulated poor prognostic leukemia.

The human ecotropic viral integration site 1 (Evi1) gene is located on 3q26.2, a region frequently rearranged in acute myeloid leukemia (AML)^1^,^2^. Most patients with 3q26 rearrangements overexpress EVI1^2^,^3^. In all, 5–10% of AML patients show Evi1 upregulation^4^. While Evi1-low patients showed >80% overall survival (OS) at 5 years, Evi1-high patients showed <60%, indicating that high expression of Evi1 correlated with poor prognosis^5^,^6^.

MicroRNAs (miRNAs) are 18–25 nt, single-stranded non-coding RNAs that are generated from primary miRNAs via pre-miRNAs. miRNAs can suppress post-transcriptional gene expression by base pairing with their target messenger RNAs (mRNAs) and inducing either translational repression or mRNA degradation^7^,^8^. miRNAs regulate a wide range of biological processes in animal development and human disease^9^,^10^.

miRNAs are promising therapeutic targets for Evi1-overexpressing AML. The advantages of miRNAs for therapy are: 1) their multiple targets; 2) low toxicity due to biological therapy; 3) and low cost due to technological innovation^11^. Evi1 deficiency severely affects not only hematopoietic stem cells but also other systems^12^. Therefore, the suppression of Evi1 is presumed to cause systemic adverse effects. miRNAs could overcome this problem because they should be expressed at an optimal dose *in vivo* under the control of endogenous feedback regulation and should affect the dysregulated overexpression of Evi1 in leukemic cells but not in other systems. Accordingly, Evi1-overexpressing leukemia should be a good target for miRNA-based therapy. In this study, we aimed to identify miRNAs that suppress Evi1 for therapeutic purposes. We found that miR-133, which targets Evi1, increased the drug sensitivity of Evi1-high-expressing leukemic cells, but not Evi1-non-expressing leukemic cells. This suggests that miR-133 is a promising therapeutic target for Evi1-overexpressing leukemia.

## Results

**Figure 1:**
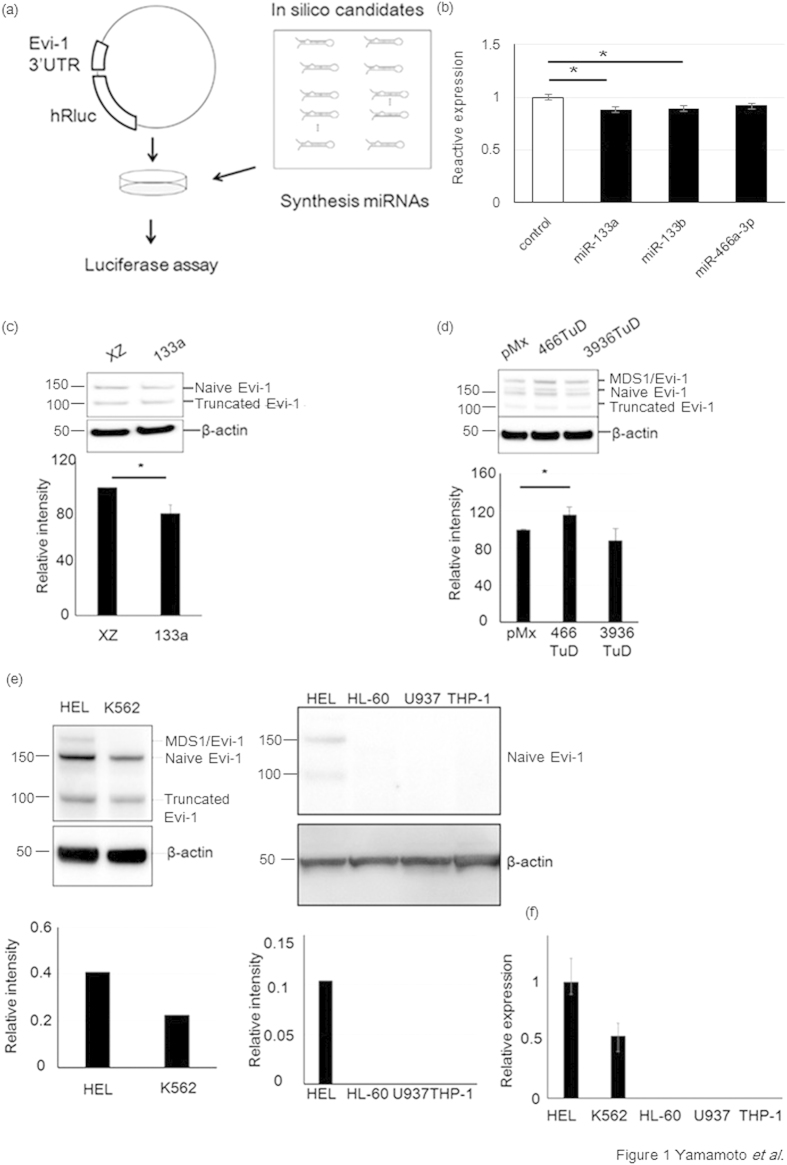
(**a**)The schema of the screening of miRNAs binding to the Evi1 3′UTR Synthetic miRNAs and the luciferase reporter containing the 3′-UTR of the human Evi1 mRNA were co-transfected into NIH3T3 cells to identify the miRNAs which downregulate luciferase by binding to Evi1 3′UTR. (**b**) The downregulated luciferase activity by binding of miR-133a,b, and miR-466a-3p to Evi1 3′UTR. The longtitude axis showed the relative luciferase to the control. (**c**) Exogenous miR-133 decreased Evi-1 expressions in HEL cells. The sum of naïve and truncated or MDS1 bands intensity was determined by densitometry and normalized to β-actin. Three experiments were done. (*p < 0.05). (**d**) In Hela cells which were transfected with miR-466 or miR-3936 tough decoy (TuD), all of Evi-1 variant proteins including MDS1/Evi-1, Evi-1 and truncated Evi-1 were specifically upregulated by miR-466 suppression. The band intensity was determined as above. Three experiments were done. (*p < 0.05). (**e**) Expression of Evi1 in HEL, K562, HL60, U937 and THP1 cells. The expression of Evi1 was high in HEL and K562 cells (Left), while that was not detected in HL60, U937 and THP-1 cells. The band intensity was determined by densitometry and normalized to β-actin. (**f**) Data of real-time PCR of expression of Evi1 in HEL, K562, HL60, U937 and THP-1 cells. Expression of Evi1 was detected only in HEL and K562 cells. (*p < 0.05)

We screened for miRNAs that potentially target Evi1 to suppress its expression using computational prediction and luciferase assays. *In silico* prediction of Evi1 targets using the miRanda software revealed that 42 miRNAs potentially bind to the Evi1 3′UTR. (Table 1) Next, we examined whether they suppress the translation of a luciferase reporter containing the 3′UTR of the human Evi1 mRNA. The precursors for 42 miRNAs were available in our miRNA precursor library. Pre-miR™ Precursor Molecules for these 42 miRNAs were co-transfected into NIH3T3 cells with a luciferase reporter vector containing the 3′UTR region of the *human Evi1* mRNA. (Figure 1a) Two miRNAs reproducibly downregulated luciferase activity: miR-133 and miR-466. (Figure 1b)

To examine whether the endogenous expression of Evi1 is affected by miR-133 and miR-466, we overexpressed miR-133 in HEL cell lines, which express high levels of Evi1. We found that miR-133 suppressed endogenous Evi1 expression in the HEL cell lines. (Figure 1c)

Overexpression of miR-466 did not affect endogenous Evi1 expression, but suppression of miR-466 activity by miR-466 TUD^13^ resulted in upregulation of endogenous Evi1 expression. (Figure 1d)

**Figure 2:**
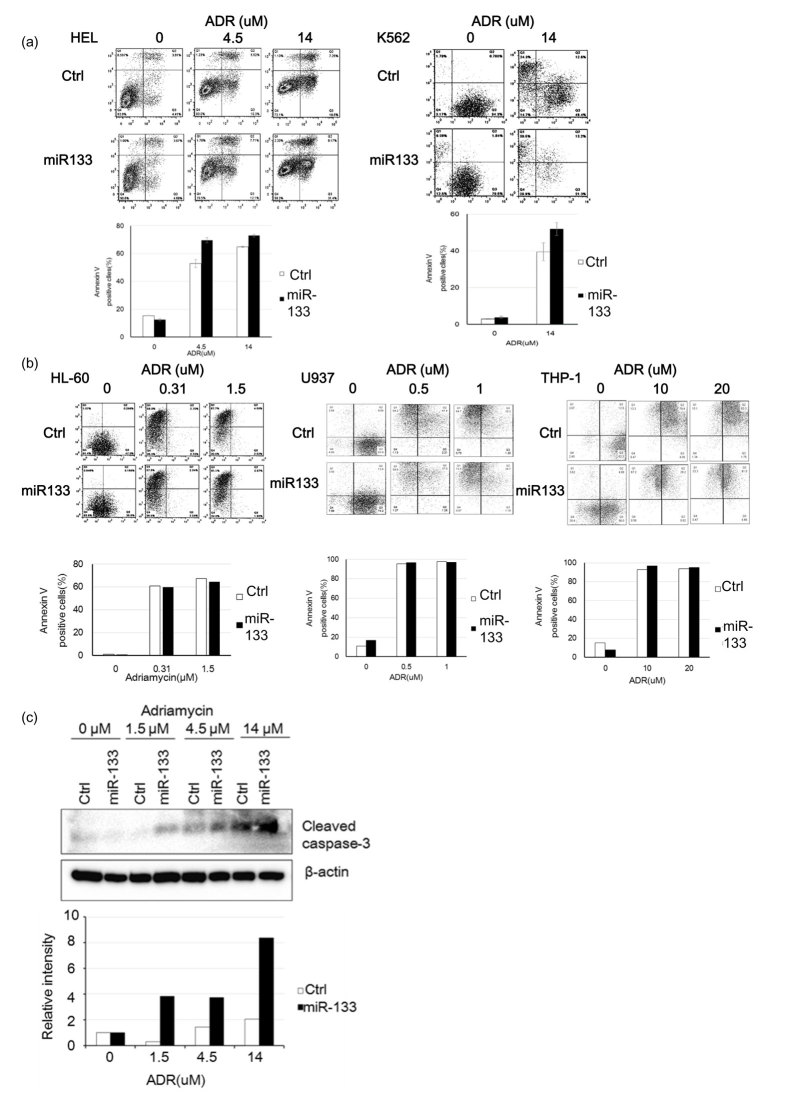
Functional Analysis for miR-133. (**a**) Exogenous expression of miR-133 in Evi-1^high^ HEL cells increases the sensitivity of ADR. Annexin V positive cells including PI positive and PI negative cells were measured by FACS analysis. miR-133 overexpressing Evi-1^high^ HEL cells and K562 cells showed more Annexin V positive cells in 4.5 and 14 μM ADR. ADR indicates Adriamycin.(*p < 005). (**b**) Exogenous expression of miR-133 in HL60 cells, U937 cells and THP1 cells had no effect on the sensitivity of ADR. Annexin V positive miR-133 overexpressing HL60 cells, U937cells, and THP-1 cells showed no difference with the control HL60 cells, U937 cells, and THP-1 cells at all the concentration of ADR. (c) Cleaved caspase 3 was increased by miR-133 in ADR dose dependent manner.

Since miR-133 and miR-466 both regulated Evi1, we further analyzed their functions in leukemia cell lines. Evi1 overexpression is associated with poor prognosis and shorter survival in AML, because AML shows strong drug resistance. We examined whether miR-133 and miR-466 increase sensitivity to Adriamycin (ADR), one of the key drugs in chemotherapy for AML. HEL and K562 cells derive from AML patients with high Evi1expression, while HL60, U937 and THP1 cells are from ones without Evi1 expression. (Figure 1e,f) We compared drug sensitivity between the two groups of cell lines. Ectopic expression of miR-133 in HEL cells sensitized the cells to ADR. HEL cells were transduced with a retroviral expression vector, MDH, expressing miR-466, miR-133, or no miRNA (control). Transduced HEL and K562 cells were sorted for those expressing GFP (a marker gene in the MDH vector) and were treated with ADR for 44 h. Annexin V staining was used to measure apoptosis. Approximately 3–7% of cells expressing miR-133 were Annexin V-positive (apoptotic) in the absence of ADR treatment, similar to sorted control MDH HEL, K562, HL60, U937 and THP1 cells. (Figure 2a,b) Treatment with ADR for 48 h dose-dependently increased the number of Annexin V-positive miR-133-overexpressing HEL and K562 cells, but not HL60, U937 and THP1 cells, compared to control cells. (Figure 2a,b) These results clearly show that miR-133 induces apoptosis in Evi1-overexpressing cells, but not in cells without Evi1 expression. miR-466 did not show any effect on ADR sensitivity.

**Figure 3:**
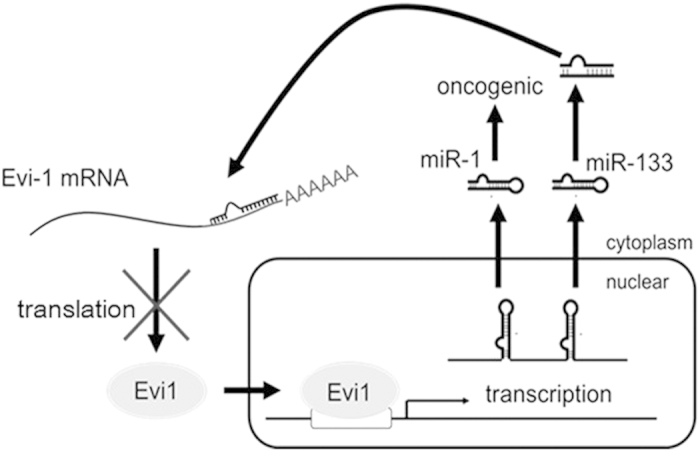
The schema of the function of miR-1 and miR-133 in Evi-1^high^ cells. Evi1 upregulates precursor of miR-1 and miR-133. While miR-1 shows oncogenic activity, miR-133 binds 3UTR of Evi1 to downregulate Evi1, which makes “negative feedback loop”. The English in this document has been checked by at least two professional editors, both native speakers of English. For a certificate, please see: http://www.textcheck.com/certificate/cTxRPr

That miR-133 increases drug sensitivity in Evi1-high-expressing AML 1 cells was confirmed by caspase activation in these cells. ADR dose-dependently increased cleaved caspase-3 levels in miR-133-overexpressing cells compared with control cells. (Figure 2c) This indicates that miR-133 promoted apoptosis in the presence of ADR in Evi1-high-expressing HEL leukemic cells. (Figure 3)

## Discussion

miR-1-2 and miR-133a-1 are clustered together at the same locus on chromosome 18^14^ suggesting that their transcription might be regulated by similar mechanisms. It was previously reported that transcription of these two miRNAs was directly regulated by Evi1, which acts as a transcription factor for them^15^.

Both miRNAs are upregulated by overexpression of Evi1, while only miR-1, and not miR-133, increased cell proliferation^16^. The function and significance of miR-133, which is transcriptionally upregulated by Evi1, needed to be explored. In this study, we demonstrated that miR-1 and miR-133 might act antagonistically, at least in Evi1-overexpressing leukemic cells. Similarly, a previous study showed that miR-1 and miR-133, which are preferentially expressed in cardiac and skeletal muscle and have been shown to regulate differentiation and proliferation of cells in these tissues, produce opposing effects: miR-1 is pro-apoptotic in cardiac cell apoptosis whereas miR-133 is anti-apoptotic. This suggests that the relative levels of miR-1 and miR-133 are more important than their absolute levels in determining the fate (apoptosis or survival) of cardiac cells. The expression difference between miR-1 and miR-133 might be governed by their biogenesis because the primary miRNA for miR-1 and miR-133 is a single transcript.

Recently, an *in silico* study showed that a SNP in the predicted miR-133 binding site in the 3′UTR of Evi1 predicted worse prognosis in AML. This suggests that in patients miR-133 may play a critical tumor suppressive role whose abrogation results in a worse prognosis^17^. Our functional assay clearly showed that miR-133 is a tumor suppressor for Evi1-overexpressing leukemic cells. The restoration of miR-133 in gastric cancer suppresses cell proliferation and induces apoptosis, indicating that miR-133 is a promising therapeutic target, consistent with our study^18^,^19^. Accordingly, regulation of miR-133 processing, chemically modified mimics of miR-133, and drug delivery systems should be further studied to better understand the function of miR-133 in Evi1-overexpressing leukemia and its therapeutic potential. Target molecules of Evi1 that induce drug resistance include ITGA6, GPR5, and ANG1^20^,^21^,^22^.

The target genes of miR-133 include MCL1, BCLxL, and IGF-1R, which have anti-apoptotic and oncogenic properties^23^,^24^,^25^,^26^,^27^. miR-133 may induce drug sensitivity through downregulation of Evi1 and these target genes.

In summary, we identified miR-133 as a miRNA that regulates Evi1, whose overexpression is associated with a poor prognosis in AML. Since miR-133 modulates dysregulated excess Evi1 expression but not normal expression, it could be a promising therapeutic target in Evi1-overexpressing AML patients.

## Materials and Methods

### Prediction of miRNAs using a computational target prediction system

To detect candidate miRNAs targeting Evi1, we first evaluated a series of miRNA precursors. To narrow the screened miRNAs to fewer than 100 miRNAs, we used a computational target prediction system (miRanda) containing updated sequences for all known miRNAs. Cutoff scores for selection of candidate miRNAs were <−20.0 for energy and >120 for binding^28^.

### Cell culture

Five cell lines (HEL, K562, U937, HL-60, and THP1) were maintained in RPMI 1640 medium (Wako, Japan) supplemented with 10% (v/v) fetal bovine serum (FBS), 50 U/mL penicillin, and 50 mg/mL streptomycin in a 10 cm dish (Corning, Inc., Corning, NY, USA). Cells were passaged twice per week.

### Quantitative PCR for genes

For target gene detection, RT-PCR was performed using the High Capacity cDNA Reverse Transcription Kit (Applied Biosystems, Inc., CA, USA) and qPCR was carried out with the Fast SYBR Green Master mix. All real-time qPCR was conducted using the StepOnePlus real-time PCR system (Applied Biosystems). Threshold cycle (CT) values were calibrated to β-actin and analyzed by the 2^−ΔΔCT^ method. Sequences of specific primers are listed in Supplementary Table 1.

### Western blotting

For Western blot analyses, cells were harvested by centrifugation and washed twice with phosphate-buffered saline (PBS). Cells (1.0 × 10^5^) were lysed in radioimmunoprecipitation assay (RIPA) buffer for 5 min on ice. Cell lysates were centrifuged to remove debris. Protein samples were separated electrophoretically on a 5–20% SDS-polyacrylamide gel and blotted onto PVDF membranes (Bio-Rad Laboratories, Tokyo, Japan). The blots were blocked with 2% low-fat dry milk in TBST (20 mM Tris-HCl, pH 7.5, 150 mM NaCl containing 0.1% Tween 20 [Sigma, MO, USA]) for 1 h at room temperature. The blocked membrane was incubated with anti-Evi1 (CST#2593) (1:2000) or anti-βactin (1:5000) for 2 h, followed by incubation with anti-rabbit IgG (CST#7074) (1:2000) secondary antibody for 1 h.

### Drug sensitivity assay

Five cell lines (HEL, K562, U937, HL-60, and THP1) were seeded in 24-well plates with 2.0 × 10^5^ cells per well in growth medium. Adriamycin was added at specific concentrations and incubated for 48 h, before being analyzed by FACS with immunostaining for APC-Annexin V (BioLegend, Japan).

## Additional Information

**How to cite this article**: Yamamoto, H. *et al.* miR-133 regulates Evi1 expression in AML cells as a potential therapeutic target. *Sci. Rep.* **5**, 19204; doi: 10.1038/srep19204 (2015).

## Supplementary Material

Supplemental Table 1

## Figures and Tables

**Table 1:** In silico predicted miRNAs which are potentially bound to the 3′UTR of human Evi1 mRNA. Candidate miRNAs.

let-7b	miR-466a-3	miR-669f-3p
let-7c	miR-466b-3p	miR-669k
miR-30c	miR-466c-3p	miR-674
miR-32	miR-466d-3p	miR-702
miR-125b-1-3p	miR-466e-3p	miR-709
miR-133a	miR-466i-3p	miR-713
miR-133b	miR-468	miR-804
miR-210	miR-470	miR-833a-5p
miR-302a	miR-471-3p	miR-833b-5p
miR-302b	miR490-5p	miR-1199
miR-302c	miR-491	miR-1894-3p
miR-302d	miR-501-5p	miR-1897-3p
miR-423-5p	miR-532-5p	miR-1934
miR-431	miR-539-5p	miR-1982.1
