# Supplementary material for: miR-133 regulates Evi1 expression in AML cells as a potential therapeutic target
Source: Sci Rep. 2016 Jan 12;6:19204. doi: 10.1038/srep19204 (PMC4709720; doi:10.1038/srep19204)
Supplement: Supplemental Table 1 [file srep19204-s1.doc]

**miR-133 regulates Evi1 expression in AML cells as a potential therapeutic target**

Haruna Yamamoto^†1^, Jun Lu^†2^, Shigeyoshi Oba^3^, Toyotaka Kawamata^4^, Akihide Yoshimi^5^, Natsumi Kurosaki^1^, Kazuaki Yokoyama^4^, Hiromichi Matsushita^6^, Mineo Kurokawa^5^, Arinobu Tojo^4^, Kiyoshi Ando^6^, Kazuhiro Morishita^7^, Koko Katagiri^8^, and Ai Kotani^*1,6,9^

^1^Department of Hematological Malignancy, Institute of Medical Science, Tokai University. 143 Shimokasuya, Isehara, Kanagawa 259-1193 Japan

^2^ Department of Intractable Diseases, Institute of National Center for Global Health and Medicine. 1-21-1 Toyama, Shinjuku-ku, Tokyo 162-8655 Japan

^3^Department of Nephrology and Endocrinology, the University of Tokyo Hospital. 7-3-1 Hongo,

Bunkyo-ku, Tokyo 113-8655 Japan

^4^Department of Hematology/ Oncology, Institute of Medical Science, University of Tokyo. 4-6-1 Shirokanedai, Minato-ku, Tokyo 108-8639 Japan

^5^Department of Hematology and Oncology, Graduate School of Medicine, the University of Tokyo. 4-6-1 Shirokanedai, Minato-ku, Tokyo 108-8639 Japan

^6^Department of Hematology / Oncology, School of Medicine, Tokai University. 143 Shimokasuya,

Isehara, Kanagawa 259-1193 Japan

^7^Department of Medical Science, Faculty of Medicine, University of Miyazaki. 5200 Kiyotakecho Kihara, Miyazaki-city, Miyazaki 889-1692 Japan

^8^Department of Biosciences, School of Science,*Kitasato*University 1-15-1, Kitasato, Minami-ku, Sagamihara, Knagawa 252-0373 Japan

^9^Japan Science and Technology Agency (JST) Science Plaza 5-3, Yonbancho, Chiyoda-ku, Tokyo 102-8666 Japan

*Correspondence: Ai Kotani, E-mail: aikotani@k-lab.jp

^†^These author equally contributed to the work.

**Supplemental Table**

**Primer of Quantitative PCR**

| Quantitative PCR | Evi1 | Forward: GACCAAGTTTTTCCTGATTTGC |
| --- | --- | --- |
|  |  | Reverse: AAATGCCTTGGGACACTGAT |
|  | βactin | Forward: CTCTTCCAGCCTTCCTTCCT |
|  |  | Reverse:AGCACTGTGTTGGCGTACAG |
